# Characteristics and management of sublingual allergen immunotherapy in children with allergic rhinitis and asthma induced by house dust mite allergens

**DOI:** 10.1186/2045-7022-4-15

**Published:** 2014-04-29

**Authors:** Florence Trebuchon, Michèle Lhéritier-Barrand, Marie David, Pascal Demoly

**Affiliations:** 1 Private Office, Montferrier sur Lez, France; 2 Stallergenes, Antony, France; 3 University Hospital of Montpellier, Montpellier, France; 4 3 Chemin du Fescau, F-34980 Montferrier-sur-Lez, France

**Keywords:** Adolescents, Allergy, Asthma, Children, House dust mite, Rhinitis, Sublingual immunotherapy

## Abstract

**Background:**

Allergen immunotherapy is a recognised intervention in patients with allergies not responding to standard pharmacotherapy or in whom pharmacotherapy is contraindicated. We describe the sublingual immunotherapy (SLIT) regimens used in children and adolescents with house dust mite (HDM) respiratory allergies in France and assess the efficacy and safety of this treatment.

**Methods:**

This was a sub-analysis of paediatric patients included in a previous retrospective, observational, multicentre study. Inclusion criteria were: age 5–17 years; respiratory allergy and proven sensitisation to HDM; at least 2 years follow-up after SLIT initiation. The following data were recorded at SLIT initiation: clinical characteristics; sensitisation profile; concomitant symptomatic medications; details of SLIT protocol. During follow-up and at the end of treatment the following data were recorded: any changes to SLIT treatment; any changes to symptomatic medications; symptom progression; adverse events. SLIT efficacy, patient compliance and satisfaction, and safety were assessed.

**Results:**

736 paediatric patients were included in this analysis. Most patients (95.5%) had allergic rhinitis, which was moderate to severe persistent in 62.8%. Allergic asthma was present in 64.0% and was mild to moderate persistent in 52.7% of these patients. The majority of patients had rhinitis with asthma (59.5%). Three-hundred and seventy five (62.3%) patients were polysensitised. Compliance was good in 86.5% of patients and SLIT was effective in 83.8%. Symptoms of rhinitis and asthma were improved in 64.6% and 64.3% of patients, respectively. A decrease in symptomatic medication was observed following SLIT initiation in patients with rhinitis and/or asthma. SLIT was well tolerated with mainly local reactions reported.

**Conclusions:**

HDM SLIT appears to be effective in children and adolescents with rhinitis and/or asthma due to HDM allergens, with no tolerability issues and similar benefits as in adults.

## Background

Allergen immunotherapy (AIT) is a recognised intervention in patients with allergies not responding to standard pharmacotherapy or in whom pharmacotherapy is contraindicated [1,2]. AIT was initially administered subcutaneously (SCIT), but this treatment is time-consuming and can be uncomfortable with frequent local adverse events such as injection site swelling [3]. Sublingual immunotherapy (SLIT) has been developed as an alternative route of administration with the aim of improving safety and tolerability. Comparisons of SLIT versus SCIT do not demonstrate superior efficacy of either administration route [4-6] although further comparative data would be valuable. In children, the convenience of home administration and tolerability of daily treatment are perhaps more important than in adults, strengthening the rationale for using SLIT as opposed to SCIT.

Recent systematic reviews of randomised, placebo-controlled trials of SLIT in adults and children show that SLIT is effective at reducing the symptoms of allergic rhinitis and asthma and is a safe route of administration [3,6-8]. Furthermore, symptomatic medication use for allergic rhinitis and asthma decreases significantly in patients who use SLIT [7-9]. These findings are consistent with the reduced use of rescue medication reported in a meta-analysis of SLIT for respiratory allergies caused by house dust mite (HDM) allergens [10]. Studies indicate that SLIT has similar efficacy in children and adults [8,11] and favourable adherence rates have been reported for SLIT in children, suggesting the “real-life” viability of this form of treatment [12,13].

The 2010 revision of the allergic rhinitis and its impact on asthma (ARIA) guidelines recommends SLIT for children with allergic rhinitis due to pollen but, because of poor evidence, SLIT was not recommended for use in children with allergic rhinitis caused by HDM [14]. Subsequent studies have reported a non-significant reduction in symptoms of allergic rhinitis and asthma in children with HDM allergies treated with SLIT compared to placebo [15,16].

In a previous report, we described the results of a retrospective, observational study on the SLIT regimens used for HDM respiratory allergies in routine practice in France and also described the treatment’s efficacy and safety [17]. Here we present a sub-analysis of the results for children and adolescents aged <18 years.

## Methods

### Study design and patients

Details of the methods used in this retrospective, observational, multicentre study have been published previously [17]. The study was carried out in 2008. Briefly, randomly selected allergy specialists collected information on 10 patients who started treatment with HDM SLIT (five starting therapy in 2002 and another five in 2005) and were monitored during treatment. Inclusion criteria were: age >5 years; respiratory allergy and proven sensitisation to HDM (positive skin test or specific IgE >0.7 kUI); at least 2 years follow-up after SLIT initiation; and detailed medical files allowing reliable and consistent data collection. Physicians completed a case report form for each patient based on the data recorded in their medical notes. As the study was retrospective in design it did not affect patient management in any way.

The study was conducted in compliance with the Declaration of Helsinki (2004), good epidemiological study guidelines published by the Association of French Speaking Epidemiologists, good Pharmacoepidemiological practice guidelines published by the International Society for Pharmacoepidemiology and local regulations.

### Treatments

The SLIT administered in this study consisted of a standardized mixture of equal proportions of *D. pteronyssinus*/*D. farinae* extract in several concentrations (0.1, 1, 10, 100 and 300 index of reactivity (IR)/ml). In most patients, the study medication was titrated incrementally up to a daily maintenance dose 300 IR over 1–2 weeks.

### Data collection

Data were collected retrospectively from the medical files of each patient. At SLIT initiation, information was collected on the patients’ clinical characteristics and sensitisation profile to the most common allergens (assessed by skin testing), any concomitant symptomatic medications being taken and details of the SLIT protocol administered. At a series of follow-up visits and at the end of treatment (or at the last visit) the following data were recorded: any changes to SLIT treatment (dose alterations, early termination, etc.); any changes to symptomatic medications; symptom progression; and any adverse events.

Treatment efficacy, patient compliance and patient satisfaction were also recorded at each follow-up visit and overall, as perceived by the physicians.

### Statistical analysis

This sub-analysis was carried out on the data from all children and adolescents (<18 years of age) included in the original study.

Descriptive statistics are presented as mean ± standard deviation (SD), or n (%).

Categorical variables were compared using the Chi^2^ test or Fisher’s exact test and continuous variables were compared using the Student’s t-test, non-parametric tests (Mann–Whitney), Kruskal-Wallis test, or analysis of variance.

All statistical analyses were performed out using SAS® software (version 8.2; SAS Institute Inc., Cary, NY, USA) and AdClin® software (version 3.1.1.; AdClin, Paris, France).

Written consent was obtained from patients/parents/legal guardians in accordance with local laws.

## Results

### Patients

A total of 736 paediatric patients who took part in the original study were included in this sub-analysis. The demographic and clinical characteristics of these patients at inclusion are summarised in Table 1. There was a slight predominance of males (63.9%) and the mean age was 10.02 ± 3.14 years.

**Table 1:** Demographic and clinical characteristics at treatment initiation

	**Total**	
	**(n = 736)**	
	**n**	**%**
**Gender**		
Male	470	63.9%
Female	265	36.1%
**Age (years)**		
Mean (±SD)	10.0 ± 3.14	
5-11 (children)	497	67.5%
12-17 (adolescents)	239	32.5%
Patients with polysensitisation*	375	62.3%
**Pathology of respiratory allergy**		
Rhinitis alone	265	36.0%
Rhinitis + asthma	438	59.5%
Asthma alone	33	4.5%
**Allergic rhinitis (with or without asthma)**		
**Disease duration (years)**		
n	703	95.5%
Mean (±SD)	3.57 ± 2.43	
Severity (ARIA)**		
Mild intermittent	57	8.1%
Moderate or severe intermittent	56	8.0%
Mild persistent	148	21.1%
Moderate or severe persistent	440	62.8%
**Allergic asthma (with or without rhinitis)**		
**Disease duration (years)**		
n	471	64.0%
Mean (±SD)	3.82 ± 2.91	
Severity (GINA 2004)**		
Intermittent	213	45.4%
Mild persistent	163	34.8%
Moderate persistent	84	17.9%
Severe persistent	9	1.9%
**Symptoms**		
***Rhinitis (with or without asthma)***		
Rhinorrhoea	625	89.4%
Sneezing	576	82.5%
Nasal obstruction	575	82.3%
Nasal pruritus	428	61.7%
Anosmia	13	1.9%
***Asthma (with or without rhinitis)***		
Cough	322	68.7%
Wheezing	245	52.1%
Difficult breathing	157	33.6%
Chest tightness	103	22.1%
***Rhinitis and/or asthma***		
Ocular pruritus	188	25.7%
Teary eyes	131	17.9%
Other	84	11.7%

Categorical variables are presented as the number and percentage of patients relative to the total population with non-missing data.

*Positive skin test to house dust mites and at least one other allergen, calculated as percentage of patients having at least one skin test.

**Missing data, n = 2 each.

The majority of patients (95.5%) had allergic rhinitis and almost two-thirds (59.5%) had both rhinitis and asthma. The mean duration of rhinitis symptoms was 3.57 years and the mean duration of asthma was 3.82 years.

### Sensitisation profile

A total of 602 patients had undergone skin testing; 375 (62.3%) of these patients were polysensitised to HDM allergens and at least one other allergen and 227 (37.7%) were monosensitised to HDM allergens only (Table 1).

The other allergens involved in polysensitisation were mainly grasses, birch pollen and animal dander.

### Clinical symptoms

The clinical symptoms of the patients at inclusion are shown in Table 1. The most common nasal symptoms were rhinorrhoea, sneezing and nasal obstruction. Rhinorrhoea and sneezing were mostly diurnal (41.8% and 46.1% of cases, respectively). Anosmia was rare (1.9%). In most of the patients with moderate or severe persistent rhinitis (83.2%), the symptoms were troublesome, causing sleep disruption and impacting on school, daily activities, hobbies and sport.

The most common respiratory symptoms were coughing and wheezing. Ocular symptoms (e.g. teary eyes, ocular pruritus) were less common than nasal or respiratory symptoms and were mostly diurnal.

### Symptomatic treatments at the time of initiation of HDM SLIT and concomitant allergen immunotherapies

At the time of SLIT initiation, oral antihistamines were prescribed for the symptomatic treatment of rhinitis to 86.9% of patients (repeat prescription in 62.5% and new prescription in 24.3%), nasal steroids were renewed in 25.7% and newly prescribed in 18.7%, and local antihistamines were renewed in 5.4% and newly prescribed in 6.1%. Treatment of patients with other medications (oral steroids, anti-leukotrienes, cromones) was uncommon (≤3% of patients each).

In patients with asthma at SLIT initiation, oral antihistamines were prescribed to 85.0% of patients (new prescription in 20.2%, repeat prescription in 64.8%) and nasal steroids were renewed in 51.3% and newly prescribed in 13.9%. Long-acting beta-agonists (LABA) and short acting beta-agonists (SABA) were used in 36.3% and 45.3% of patients respectively (mainly as renewed prescriptions 27.8% and 38.7%, respectively). Anti-leukotrienes were given to 15.8% of patients. Other treatments for asthma (cromones, oral LABA, oral corticoids, theophylline) were uncommon (<2% of patients each).

Concomitant AIT (in addition to HDM SLIT) was given to 150 of the 736 (20.4%) children. The main allergens involved were grasses (98/150; 65.3%), birch tree pollen (15/150; 10.0%) and animal dander (13/150; 8.7%).

### Treatment regimens

The SLIT treatment regimens used in the initiation phase comprised daily dosing in 95.6% of patients (629/658). The mean maintenance dose was 1016.95 ± 413.79 IR/week (n = 521) and the mean time taken to reach the maintenance dose was 3.9 ± 5.1 weeks (n = 655). In 38.8% of patients, SLIT treatment was still ongoing on the day of final data collection and in 61.2% treatment had been stopped, in most cases (54.9%) because the end of the treatment period had been reached during the study. The median duration of treatment was 3.1 years.

### Efficacy

**Figure 1:**
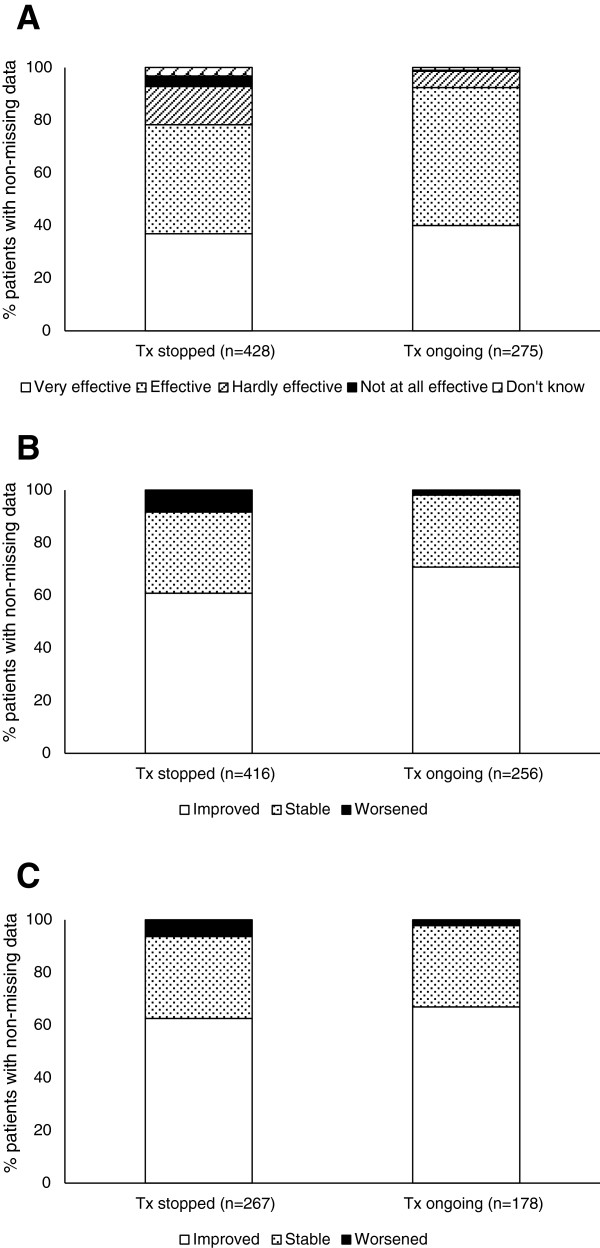
**Physician-perceived efficacy of house dust mite SLIT at the last recorded visit. A**. Physicians’ overall evaluation of SLIT / **B**. Symptoms of rhinitis / **C**. Symptoms of asthma.

Physicians considered SLIT to be effective in 83.8% of patients (Figure 1). Symptoms of rhinitis were considered to be improved in the majority of patients (64.6%) with or without asthma and symptoms of asthma were improved in 64.3% (Figure 1).

### Changes in symptomatic medication use during treatment

**Figure 2:**
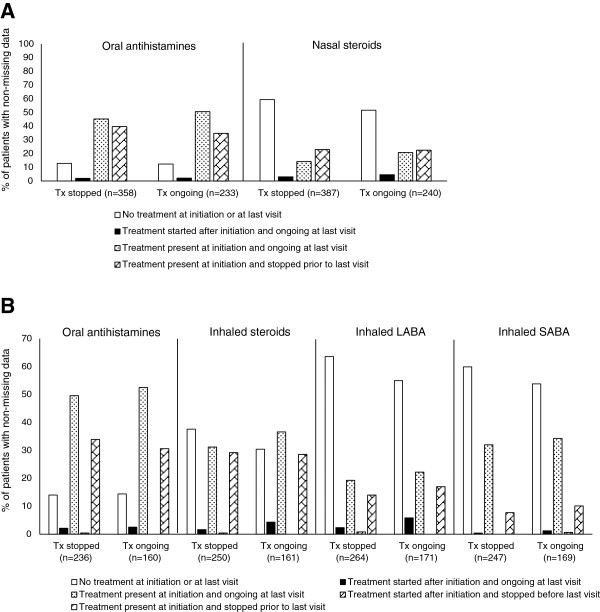
**Change in symptomatic medication use during SLIT. A**. Symptomatic treatments for allergic rhinitis / **B**. Symptomatic treatments for asthma.

Symptomatic medication use for both rhinitis and asthma decreased after SLIT initiation. Over one-third (223/591; 37.7%) of patients with rhinitis who received a prescription (new or repeat) for oral antihistamines at SLIT initiation stopped this treatment before the last visit and 22.8% (143/627) stopped using nasal steroids (Figure 2). The percentages were similar in patients in whom SLIT had been stopped or was ongoing at the time of data collection.Among the patients with asthma, 32.6% (129/396) of those newly prescribed or given a repeat prescription of oral antihistamines at SLIT initiation stopped using this medication before the last visit. This figure was 29.0% (119/411) for inhaled steroids, 15.2% (66/435) for LABA, and 8.7% (36/416) for SABA (Figure 2). This profile was similar in patients in whom SLIT had been stopped and in those in whom SLIT was ongoing at the end of the study.

### Compliance

**Figure 3:**
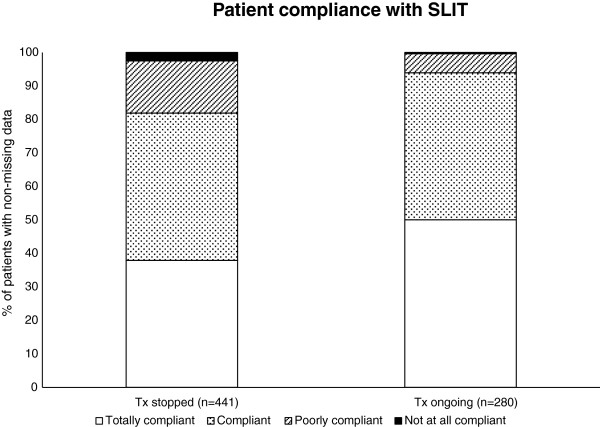
Patient compliance with house dust mite SLIT as perceived by the physicians.

Compliance was deemed to be good or very good by the physicians in 86.5% of patients overall. This percentage was higher among patients in whom treatment was still ongoing at the end of the study period (93.9%) than among those whose treatment had been stopped (81.9%) (Figure 3).

### Satisfaction

**Figure 4:**
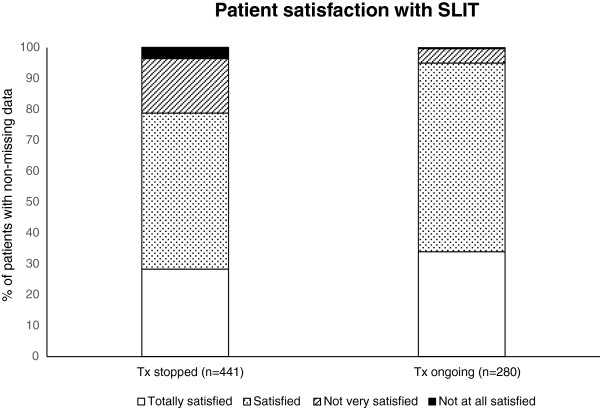
Patient satisfaction with house dust mite SLIT as perceived by the physicians.

The majority of patients overall (85.1%) were perceived by the physicians to be satisfied or very satisfied with their treatment (Figure 4). This included 78.8% of patients who had stopped SLIT for any reason and 95% of patients whose treatment was still ongoing at data collection. There was no significant difference in the levels of satisfaction between children aged 5–11 years and adolescents aged 11–17 years (79.8% vs. 76.7%, respectively, of patients in whom treatment was stopped were satisfied or very satisfied with treatment; 94.4% vs. 96.4%, respectively, in whom treatment was ongoing).

### Safety

SLIT was well tolerated (Table 2). Only 49 of the 275 patients for whom data were available reported an adverse event during a follow-up visit (17.8%). These were local reactions in the majority of cases (41/46; 89.1%) (Table 2).

**Table 2:** Tolerance to house dust mite SLIT during treatment

**Patients with at least one follow-up visit**	**(n = 278)**
Patients with at least one adverse event reported during a follow-up visit*	
No	226 (82.2%)
Yes	49 (17.8%)
If yes**	
At least one local reaction reported	41 (89.1%)
At least one systemic reaction reported	7 (15.2%)
At least one other reaction reported^#^	1 (2.2%)

*Data missing, n = 3; **data missing, n = 3; ^#^mainly pruritus.

## Discussion

This study shows that HDM SLIT is effective in the majority of children and adolescents with respiratory allergies caused by HDM allergens, with high rates of patient satisfaction, particularly among those still receiving treatment. Symptoms of rhinitis and asthma were improved in almost two-thirds of patients. Consequently, there was a significant decrease in the use of symptomatic medications, confirming the findings of previous reports [7-9]. Compliance was also high showing that treatment was acceptable to both patients and their parents.

There appears to be little difference between paediatric patients and adults in this setting since the percentage of children and adolescents who were polysensitised to HDM plus at least one other allergen was very similar to that of the adult patients included in the original study (Table 3). Furthermore, SLIT efficacy, satisfaction and compliance were also very similar in adults and children (Table 3), indicating that the benefits of HDM SLIT are similar in children, adolescents and adults. In children/adolescents as in adults [17], the mean target maintenance dose of HDM SLIT was <150 IR/day. The achievement of efficacy at such low doses may help explain the favourable tolerability of this therapy.

**Table 3:** **Data for children and adolescents compared to those for adult patients included in the original study **[17]

	**Children/adolescents**	**Adults**
	**(N = 736)**	**(N = 551)**
Rhinitis	95.5%	98.2%
Asthma	64.0%	41.0%
Rhinitis + asthma	59.5%	39.0%
Rhinitis duration (years)	3.57 ± 2.43	8.97 ± 7.84
Asthma duration (years)	3.82 ± 2.91	8.75 ± 8.38
Polysensitisation	62.3%	62.8%
***House dust mite SLIT***		
Compliance	86.5%	90.9%
Efficacy	83.8%	80.9%
Satisfaction	85.3%	86.4%

It is interesting to note that although the percentage of children and adults with rhinitis was similar, considerably more children than adults had rhinitis with asthma (59.5% vs. 39%, respectively) (Table 3). Unsurprisingly, the mean duration of rhinitis with or without asthma at the time of SLIT initiation was shorter in children than in adults and this was also the case for asthma (Table 3). However, there was no evidence that the severity of symptoms of either disease was significantly different in children compared to adults.

The present results reflect the findings of previous studies indicating similar efficacy of HDM SLIT in children and adults [3,11]. High rates of patient satisfaction coupled with high levels of compliance (median duration of treatment 2.9 years) indicate that SLIT is highly acceptable and that there are no practical difficulties with administering the treatment as prescribed. Although a universally accepted system to grade and classify the severity of adverse events of SLIT has only been proposed recently [18] and was not available when this study was carried out, the low incidence of adverse events reported (17.8%) and their mild and local nature indicate a lack of tolerability issues, supporting the high degree of patient acceptability. These aspects are particularly important among children where compliance is a major determinant for allergy treatment, especially when managed at home. In 2007, a study of simplified once-daily SLIT in children reported that 84% and 66% of subjects had compliance rates >75% and >90% at 6 months, respectively, compared with 85% and 69% at 3 months [13]. A systematic review of compliance with SLIT, published a year later, found compliance rates ranging between 75% and 90% [19]. The main causes of non-compliance were inconvenience and the cost of treatment [19]. The proportion of children in our study reported to have good or very good compliance (86.5%) is consistent with previous results. Overall, the available data show that children’s compliance with SLIT is good, even in “real-life” settings with long-term treatment administered at home.

### Strengths and limitations

Our study has some limitations. First, it was retrospective in design and was uncontrolled leading to a potential bias in the results. Furthermore, the study population was highly heterogeneous in terms of age, symptoms at SLIT initiation, symptomatic medication use, etc., due to its “real-life” setting and observational nature. Finally, the study was not originally designed to compare the efficacy and safety of SLIT in children (5–11 years) vs. adolescents (11–17 years) or to compare these different age groups with adults and some data are therefore missing.

The strengths of the study include the large number of patients included in the analysis (n = 736) and the fact that it was carried out in a “real-life” setting, in the medical surgeries of French allergy specialists. Finally, the long duration of the study, from 2002 (first patient inclusion) until data collection in 2008, enabled long-term treatment (median duration 2.9 years), follow-up and assessment of SLIT efficacy and tolerance in the children and adolescents.

This study adds to the growing evidence that SLIT is effective in paediatric patients with respiratory allergies. For example, a recent review found high-quality evidence of medication reduction and symptom control in children with asthma treated with HDM SLIT [20]. The mechanism of action of SLIT has long been believed to involve modulation of the local immune response to allergens, changing the response from an allergic reaction to immune tolerance [21]. This is attributable to the specific biology of oral antigen-presenting cells [22]. Langerhans cells, myeloid dendritic cells and macrophages in oral tissues may, in the absence of danger signals, induce CD4+ regulatory T-cells which support tolerance [22]. Induction of anti-inflammatory immunoglobulin G and immunoglobulin A also appears to play a role. The present data, acquired over a long treatment period, suggest that the effects of SLIT do not diminish over time and are not dependent on the patient’s age.

## Conclusions

This sub-analysis of the results from our original study [17] indicates that HDM SLIT may confer similar benefits in children and adolescents as in adults. Our results show good efficacy with HDM SLIT, high patient acceptability and no tolerability issues among paediatric patients. These findings reflect the results of previous investigations and support trials of SLIT in children and adolescents who have failed to respond to standard pharmacotherapy or in whom pharmacotherapy is contraindicated.

## Abbreviations

AIT: Allergen immunotherapy; ARIA: Allergic rhinitis and its impact on asthma; HDM: House dust mite; IR: Index of reactivity; LABA: Long-acting beta agonist; SABA: Short-acting beta agonist; SCIT: Subcutaneous immunotherapy; SD: Standard deviation; SLIT: Sublingual immunotherapy.

## Competing interests

F Trebuchon has no competing interests to declare. M Lheritier-Barrand and M David are employees of Stallergenes. P Demoly is a consultant and speaker for Stallergenes, ALK and Therabel, and has been a speaker for Schering-Plough-MSD, AstraZeneca and GlaxoSmithKline.

## Authors’ contributions

FT and PD were study investigators. ML-B and MD contributed to the study design and data analysis. All authors read and approved the final manuscript.
